# Did angiodysplasia associated with heyde’s syndrome disappear spontaneously?: a case report

**DOI:** 10.1186/s13019-023-02337-8

**Published:** 2023-07-10

**Authors:** Rui Li, Shuliang Ji, Jiaxi Shi, Lijin Qing, Wei Wu, Jiechun Zhang

**Affiliations:** 1 Department of Cardiovascular Medicine, General Hospital of Southern Theater Command of PLA, Guangzhou, China; 2 Department of Traditional Chinese Medicine, Shantou University Medical College, Shantou, China; 3 Guangzhou University of Chinese Medicine, Guangzhou, China; 4 Department of Cardiovascular Medicine, The First Affiliated Hospital of Guangzhou University of Chinese Medicine, Guangzhou, China; 5 The affiliated TCM Hospital of Guangzhou Medical University, Guangzhou, China

**Keywords:** Heyde’s syndrome, Aortic valve stenosis, Gastrointestinal bleeding, von Willebrand factor, Case report

## Abstract

**Background:**

Heyde’s syndrome can be easily overlooked or misjudged in clinical practice because it shares common clinical manifestations with multiple diseases as well as limited accuracy of several corresponding examinations for diagnosing Heyde’s triad. Moreover, aortic valve replacement is often delayed in these patients due to the contradiction between anticoagulation and hemostasis. Herein, we present a rare case of atypical Heyde’s syndrome. The patient’s severe intermittent gastrointestinal bleeding was not completely cured even through a local enterectomy. In the absence of direct evidence of acquired von Willebrand syndrome (AVWS) or angiodysplasia, her long-standing gastrointestinal bleeding was finally stopped after receiving transcatheter aortic valve implantation (TAVI).

**Case presentation:**

A 64-year-old female suffered from refractory gastrointestinal bleeding and exertional dyspnoea. A local enterectomy was performed owing to persistent hemorrhage and repeated transfusions; subsequently, histological examination revealed angiodysplasia. Heyde’s syndrome was not suspected until 3 years later, at which time the patient started bleeding again and was also found to have severe aortic valve stenosis upon echocardiography. TAVI was consequently performed when the patient was in a relatively stable condition even though the predisposition to bleed, but there was no evidence of angiodysplasia and AVWS during angiography at that time. The patient’s above symptoms were significantly relieved after TAVI and followed up for 2 years without any significant ischemic or bleeding events.

**Conclusions:**

The visible characteristics of angiodysplasia or a shortage of HMWM-vWFs should not be indispensable for the clinical diagnosis of Heyde’s syndrome. Enterectomy could be a bridging therapy for aortic valve replacement in patients with severe hemorrhage, and TAVI may be beneficial for moderate to high surgical-risk patients even if they have a potential risk of bleeding.

**Supplementary Information:**

The online version contains supplementary material available at 10.1186/s13019-023-02337-8.

## Background

The incidence and mortality of Heyde’s syndrome have been on the rise for nearly a decade [1]. The most convincing pathophysiological mechanism of this syndrome is acquired deficiency in high-molecular-weight multimers of von Willebrand factor (HMWM-vWFs). Despite the lack of guidelines for Heyde’s syndrome, the diagnosis is generally based on the triad of aortic stenosis (AS), refractory gastrointestinal bleeding (GIB), and associated angiodysplasia or acquired von Willebrand syndrome (AVWS) [2]. However, since it exhibits similar clinical manifestations to many other diseases, particularly gastrointestinal disorders, as well as the corresponding diagnostic examinations have limited accuracy, it is easy to be omited or misdiagnosed. Moreover, aortic valve replacement is often delayed due to the contradiction between anticoagulation and hemostasis [3]. Herein, we summarize a case of Heyde’s syndrome in which the patient’s severe intermittent gastrointestinal bleeding was not completely cured even after intestinal resection. However, transcatheter aortic valve implantation (TAVI) could dramatically improve recurrent bleeding without any bleeding or ischemic events, although angiodysplasia or AVWS could not be proven finally.

## Case presentation

A 64-year-old woman had been experiencing frequent chest distress and shortness of breath during moderate activity. She underwent two surgeries to remove breast cancer and received corresponding radiation and chemotherapy treatments 10 years ago, as well as suffered from hypertension for nearly a decade.

**Fig. 1:**
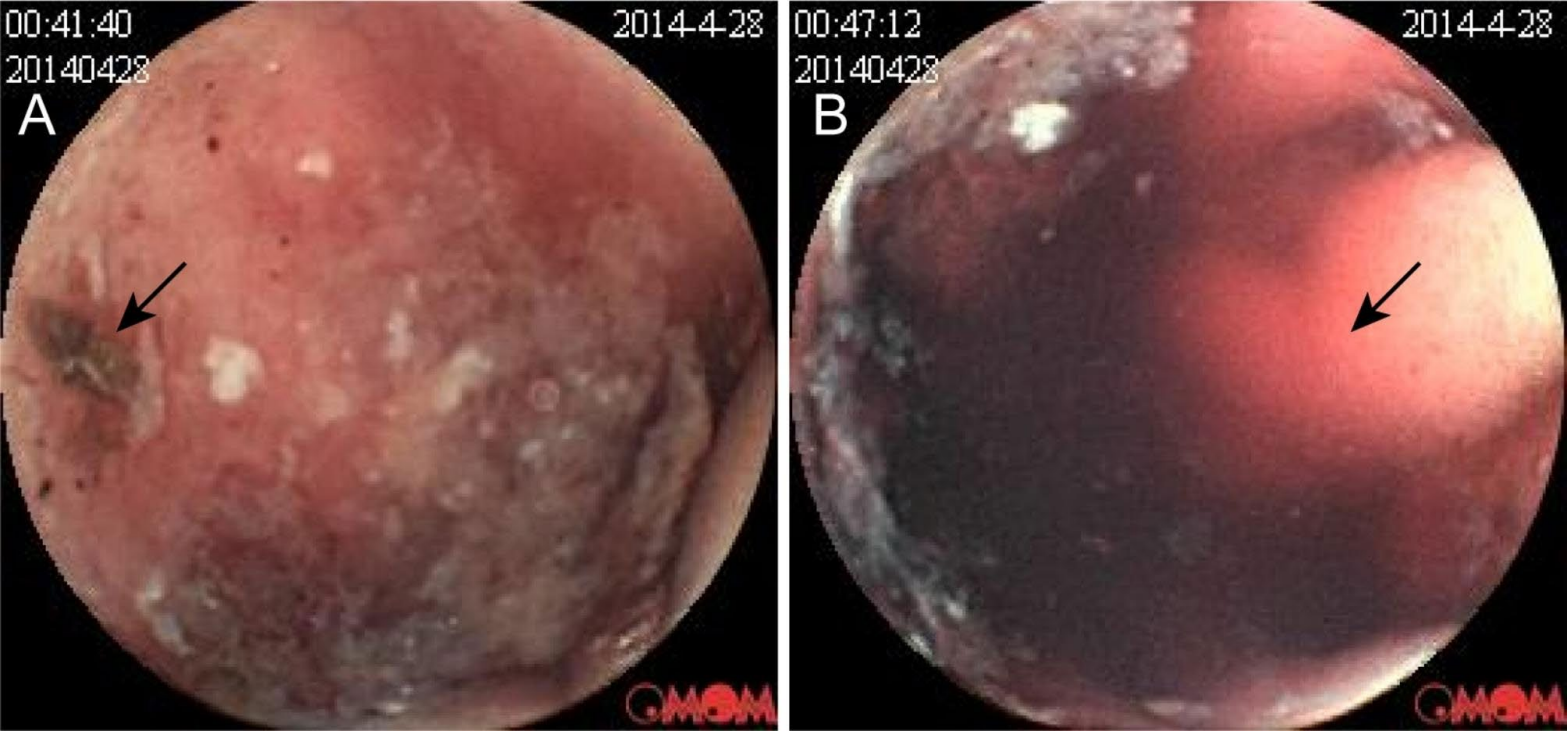
Pictures of the patient’s capsule endoscopy before laparotomy. Capsule endoscopy pictures show blood scabs(A), active bleeding(B), and hematoma formation(B) in the jejunum (indicated by black arrows)

In February 2014, she repeated episodes of tarry stools accompanied by palpitations, chest tightness, and dizziness without any apparent inciting factors. Gastroduodenoscopy and colonoscopy did not locate any bleeding lesions, while subsequent capsule endoscopy and double balloon enteroscopy identified hemorrhage and multiple haemangiomas in the jejunum respectively at local hospitals (Fig. 1).

Nevertheless, there was minimal hemostatic effect following endoscopic argon plasma coagulation and conventional medication. She then had to receive a transfusion of several units of blood, during which her hemoglobin fluctuated between 74 and 124 g/L (normal 110–150 g/L). Several months later, as she required repeated transfusions due to her persistent hemorrhage, an exploratory laparotomy was performed to resect several suspicious lesions in her small intestine for further diagnosis and treatment. The relevant histological examination showed chronic mucosal inflammation with multiple erosions, which was consistent with a haemangioma. Thus, thalidomide (25 mg/d) was prescribed to prevent postoperative neoangiogenesis, and her GIB consequently ceased for some time.

In May 2017, the patient received an emergency coronary angiography because of fainting during exercise. The coronary angiography revealed a diffuse stenosis of 60% in the middle right coronary artery but no other critical lesions. Interestingly, probably limited by the expertise of the local hospital, the echocardiogram report only mentioned mild AS and aortic regurgitation. The patient started having melena again after the procedure, which was likely related to the dual antiplatelet therapy before coronary angiography. However, the GIB was difficult to be completely controlled by conventional medications, such as proton pump inhibitors (PPIs) and octreotide.

**Fig. 2:**
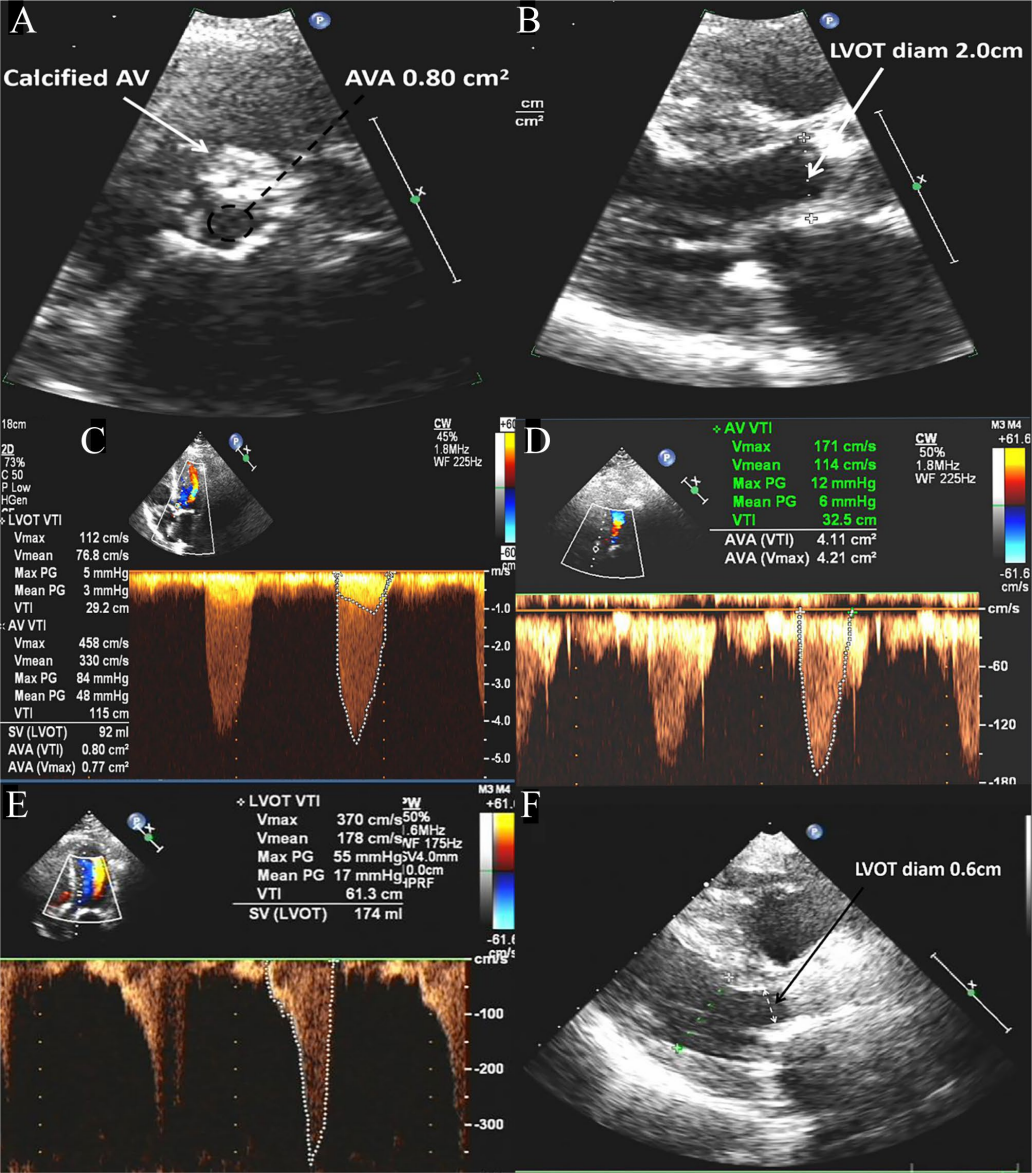
Pictures of the patient’s Ultrasound Cardiogram. Preprocedural TTE shows (**A**) severe AS with a calcified aortic valve (AV) and an aortic valve area (AVA) of 0.80 cm2; (**B**) the left ventricular outflow tract (LVOT) diameter was 2.0 cm; and (**C**) the maximum peak gradients in the LVOT and AV were 5 mmHg and 84 mmHg, respectively; the maximum systolic flow velocities of the LVOT and AV were 1.12 m/s and 4.58 m/s, respectively. TTE at the one-year follow-up shows (**D, E**) that the maximum peak gradients in the LVOT and AV were 55 mmHg and 12 mmHg, respectively; the maximum systolic flow velocities in the LVOT and AV were 3.70 m/s and 1.71 m/s, respectively; the AVA was 4.11 cm2; and (**F**) the LVOT diameter was 0.60 cm

Subsequently, the patient was referred to our department due to worsening exertional dyspnoea, thoracalgia, and orthostatic hypotension. Her clinical signs of AS and systemic congestion were obvious, the laboratory tests were unremarkable other than a Hb level of 76 g/L and an NT-proBNP level of 392 (normal < 125) pg/mL. The transthoracic echocardiography (TTE) results showed severe tricuspid calcified AS, the mean and peak transvalvular pressure gradients were 48 and 84 mmHg, respectively; the aortic valve area was 0.80 cm2; and there were no signs of obstructive hypertrophic cardiomyopathy (HCM) (Fig. 2a-c).

**Fig. 3:**
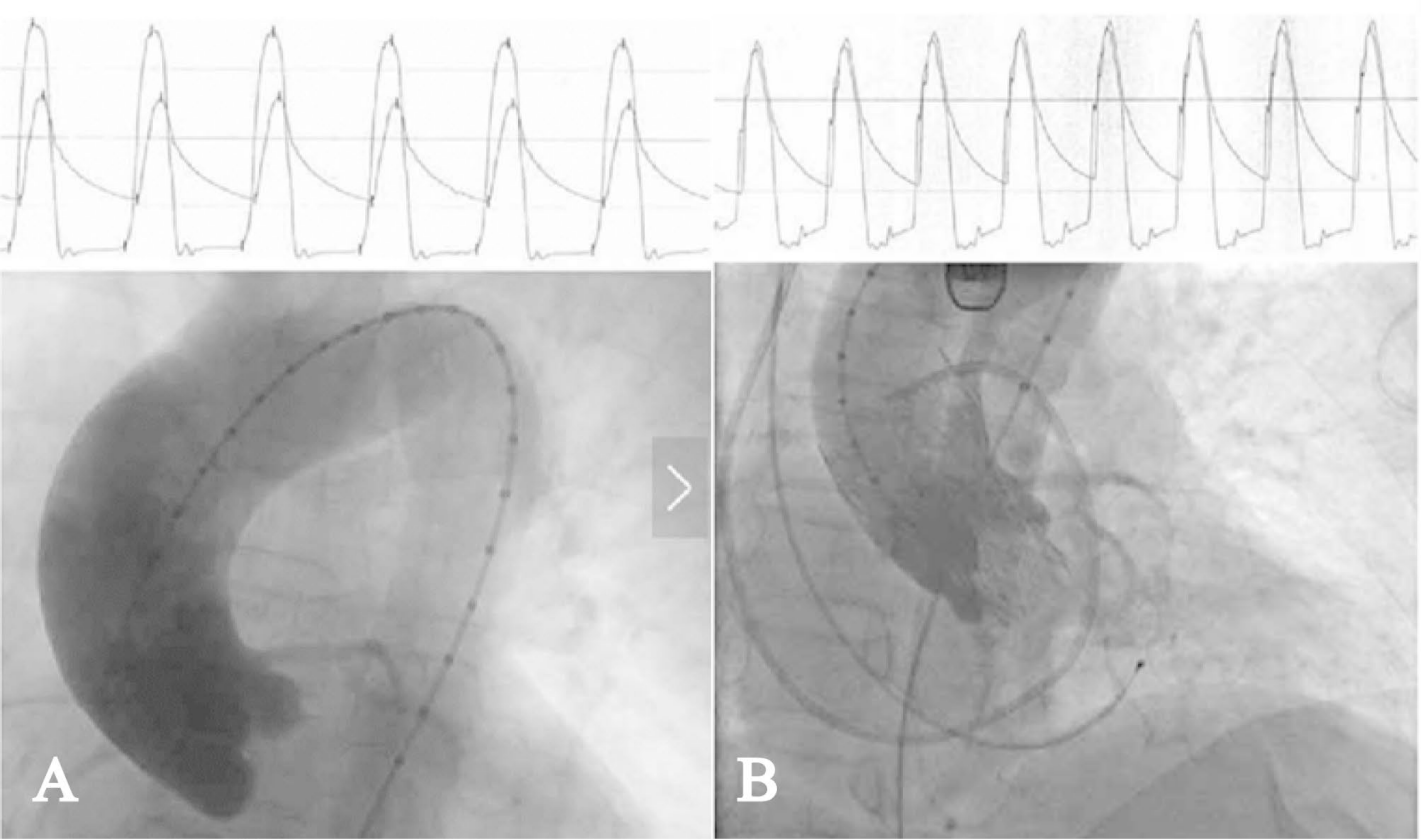
Pressure gradient charts across the aortic valve before and after TAVI. (**A**) Transvalvular pressure gradient is 88mmHg before TAVI;(**B**) Transvalvular pressure gradient is lower than 5mmHg after TAVI

After comprehensively understanding the overall condition of the patient through the Heart Team and taking into full consideration her own wishes, the patient ultimately underwent TAVI with a VENUS-A 26 prosthetic aortic valve (Venus Medtech (Hangzhou) Inc.) with no paravalvular leak occurred after the procedure in August 2018. Interestingly, arteriovenous malformations are difficult to detect via superior mesenteric artery angiography, and there was still a 60% stenosis in the mid-segment of the right coronary artery through coronary angiography before TAVI. The patient’s peak aortic valve pressure gradient decreased rapidly from 88 mmHg to less than 5 mmHg postoperatively, and she did not develop enterorrhagia or major bleeding during the perioperative period (Fig. 3). For postoperative anti-thrombotic therapy, clopidogrel monotherapy was taken lifelong accompanied by dabigatran for 6 months.

At the 2-year follow-up, the TTE results confirmed a well-seated prosthesis with a peak transvalvular pressure gradient of 12 mmHg and an aortic valve area of 4.11 cm2, but there was an isolated left ventricular outflow tract obstruction (LVOTO) present, which was not accompanied by a systolic anterior motion of the mitral valve or HCM (Fig. 2d-e). She did not suffer any further significant hemorrhagic or ischaemic events or symptoms secondary to the triad of AS.

## Discussion

### Epidemiology and pathophysiology

With the aging of society, the prevalence of Heyde’s syndrome in patients with severe AS is increasing annually and has reached 1.87-3.2%, resulting in increasing disease mortality [1]. The corresponding dominating mechanism is the excessive proteolysis of HMWM-vWFs by ADAMTS-13 in the context of the high shear stress related to stenotic valves, which not only predisposes patients to bleed but also induces submucosal arteriovenous malformations in the digestive tract [4, 5].

## Diagnosis

It is generally believed that the diagnosis of Heyde’s syndrome should be based on a triad of AS, refractory GIB, and associated angiodysplasia or AVWS. Additionally, other primary diseases that can cause GIB should be excluded, such as tumors; primary digestive, hematological, or autoimmune diseases; and the side effects of drugs.

For AS, the risk of complicating haematochezia in patients with severe AS is known to be 100 times of that in non-AS patients [6]. As the patient suffered from continuous dyspnoea, chest pain, palpitations, and even fainting during mild to moderate exercise, the diagnosis of AS should be recognized earlier followed by excluding severe coronary heart disease. Combined with her symptoms of GIB, it should be further differentiated from Heyde’s syndrome promptly.

For angiodysplasia, locating the culprit lesion can be challenging. Despite the rapid development of endoscopic diagnostic technologies in the past decade, including double balloon enteroscopy, 35% of angiodysplasia cases have gone undiagnosed [3]. Although the gold standard diagnostic method is mesenteric arteriography, the average localization rate of the bleeding site is 40% since many cases of bleeding angiodysplasia only intermittently bleed, which decreases its sensitivity for detection [7]. The angiodysplasia of the patient was confirmed by histological examination through local enterectomy, but angiodysplasia was not observed via mesentericography several years later. The phenomenon might be primarily attributed to the observable suspicious lesions in the gut that had been surgically removed or were restricted by thalidomide, and the patient was relatively stable when she underwent mesentericography.

For AVWS, the necessity of gel electrophoresis to confirm the loss of large multimers also makes the biological diagnosis of Heyde’s syndrome challenging. Coagulopathy may be absent in patients with aortic gradients below 50 mmHg, and the corresponding testing is costly and time-consuming [2]. The vWFs were not examined for the patient due to the limited capabilities of our laboratory department. Research shows that the prevalence of abnormal HMWM-vWFs in patients with native AS is estimated to be 65–92%, and the incidence of bleeding angiodysplasia in patients with AVWS is approximately 11.5%. Furthermore, 55.6–87.5% of patients with documented angiodysplasia have a deficiency in HMWM-vWFs [8]. However, other studies indicate no increase in the prevalence of AVWS in patients with bleeding angiodysplasia [9]. In the context of AS, other mechanisms, such as low perfusion, submucosal ischemia, hypoxia, or cholesterol embolism, have also been considered to be the cause of the relationship between angiodysplasia, GIB, and AS [10].

We searched for case reports of Heyde’s syndrome that were published since 2000 in PubMed and obtained 91 articles. 13 articles that without available relevant information (n = 10), inconsistent with the disease (n = 1), described a patient with epistaxis (n = 1), and described an infant case (n = 1) were excluded. The remaining 78 articles involving a total of 83 cases were summarized (5 articles were double-case reports) with special references to the treatment methods, angiodysplasia, and HMWM-vWFs (Additional Table 1). Among these case reports, the primary diseases were severe LVOTO in 6 patients, severe aortic regurgitation in 1 patient, and AS in the remaining 76 patients; 17 of these patients (20.5%) were not diagnosed with angiodysplasia, of which 11 (84.6%) of the 13 patients who were undergoing cardiac surgery (including aortic valve replacement and alcohol septal ablation) were cured of GIB. Additionally, 9 patients (10.8%) had no deficiencies in HMWM-vWFs; 46 patients (55.4%) did not get tested for HMWM-vWFs, of which 31 (91.2%) of the 34 patients who had cardiac surgery had GIB remission. 10 patients were diagnosed with neither angiodysplasia nor AVWS, of which 6 (85.7%) of the 7 patients who underwent heart surgery were cured of GIB.

Given the complicated mechanisms of Heyde’s syndrome and the imperfect accuracy of available relevant diagnostic methods, we should not simply exclude Heyde’s syndrome in practice merely due to a lack of evidence for the presence of bleeding angiodysplasia or AVWS. Notably, we should consider the feasibility of Heyde’s syndrome as an exclusionary diagnosis and aortic valve replacement as a diagnostic therapy.

### Treatment

Endoscopic therapies are often ineffective, whereas local enterectomy could be a bridging therapy for aortic valve replacement in patients with continuous enterorrhagia or major or life-threatening hemorrhage. Nevertheless, the bleeding recurrence rate after intestinal resection is approximately 30%, which is mainly due to angiodysplasia that cannot be cured locally [11]. The patient underwent segmental enterectomy for severe bleeding which was only temporarily alleviated followed by regular administration of the angiogenesis inhibitor thalidomide after surgery.

SAVR or TAVI is recommended to be a radical therapy for Heyde’s syndrome [12]. In comparison, TAVI may be not only an optimal modality for high-risk patients, such as patients who are elderly, have multiple comorbidities, or have a hemorrhagic predisposition, but also suitable for patients with intermediate to low surgical risk. Van et al. [13] conducted a randomized controlled trial in 87 canters, including 1,660 patients with severe aortic valve stenosis and moderate surgical risk. The 5-year follow-up showed that the incidence of mortality or disabling stroke after TAVI was similar to that of patients undergoing SAVR (31.3% vs. 30.8%; *P* = 0.85), also with a lower transvalvular pressure gradient (8.6mmHg vs. 11.2 mmHg; *P* < 0.001) and larger aortic valve area (2.2 cm^2^ vs. 1.8 cm^2^; *P* < 0.001). Although the incidence of the paravalvular leak was higher after TAVI than in patients who underwent SAVR (3.0% vs. 0.7%; *P* = 0.05). A randomized controlled trial including 1,403 patients with severe aortic valve stenosis and low surgical risk showed that there was no significant difference in the incidence of all-cause mortality or disabling stroke between the TAVI and SAVR groups at 24 months (5.3% vs. 6.7%, P > 0.999). Additionally, the TAVI group had a lower incidence of disabling stroke, bleeding complications, acute kidney injury, and atrial fibrillation within 30 days compared to the SAVR group, although the incidence of moderate or greater aortic valve regurgitation (3.5% vs. 0.5%) was relatively higher in the TAVI group [14]. Desai et al. [1] found no significant differences in all-cause mortality or total expenses during hospitalization between TAVI and SAVR. Moreover, TAVI is superior to SAVR in lessening the duration of hospitalization and the incidence of periprocedural complications such as stroke, myocardial infarction, or major or life-threatening bleeding.

Nevertheless, patients who had TAVI might be prone to complicate with paravalvular regurgitation (26.6% vs. 4.2%, P < 0.001) which is independently associated with the risk of re-bleeding(OR, 3.65 [95%CI, 1.36–9.80];*P* = 0.01), and higher incidence of late-stage GIB (3.3% vs. 1.5%, P < 0.001) than SAVR [12, 15, 16]. In addition, an observational study including 890 patients undergoing transcatheter or surgical bioprosthetic aortic valve replacement found that the incidence of subclinical leaflet thrombosis following TAVI was significantly higher than after SAVR (13% vs. 4%, *P* = 0.001) in a median follow-up period of 83 days. However, the incidence of subclinical leaflet thrombosis in patients taking anticoagulant medications (including warfarin and NOACs) after the procedure was much lower than in those receiving dual or single antiplatelet therapy (4% vs. 15%, *P* < 0.0001; 4% vs. 16%, *P* < 0.0001). There was no significant difference between NOACs and warfarin in preventing subclinical leaflet thrombosis (3% vs. 4%, *P* = 0.72), while subclinical valve thrombosis was significantly associated with postoperative TIA or stroke (7.85% vs. 2.36%, *P* = 0.001) [17].

Taking into consideration the patient’s history of chest radiation therapy, recurrent GIB, mild to moderate anemia, poor cardiac function, and STS score of 4.052%, as well as the patient’s unwillingness to undergo surgical intervention, the Heart Team has made a cautious decision to perform TAVI to avoid surgical complications and increase the benefit of valve replacement. To prevent post-TAVI leaflet thrombosis and its potential consequences of stroke as well as treatment of coronary heart disease, and based on the recommendations of the 2017 AHA/ACC guidelines for valvular heart disease [18], the patient was administered dabigatran combined with clopidogrel for 6 months after TAVI, whereas the latest relevant guidelines no longer recommend the use of any anticoagulants in patients without indications for anticoagulation after TAVI [19].

Although some cases of Heyde’s syndrome are caused primarily by LVOTO, we are not looking for any prognostic reports concerning the complication of LVOTO after TAVI in patients with Heyde’s syndrome. One study indicated that 89% of patients with baseline or latent obstructive HCM suffered abnormalities in HMWM-vWFs when the peak gradient of the obstructive LVOT was > 15 mmHg (at rest) or > 35 mmHg (during exercise). Additionally, approximately 30% of those patients developed GIB [20, 21]. The patient, who was verified as having no myocardial hypertrophy or LVOTO by preoperative echocardiography, was found to be complicated with an asymptomatic and isolated LVOTO (maximum peak gradient in LVOT of 55 mmHg) at the 1-year follow-up. It is worth further investigating whether and how the LVOTO following TAVI in patients with Heyde’s syndrome impacts their prognosis regarding GIB.

## Conclusion

Elderly patients suffering from bleeding angiodysplasia and refractory GIB due to unknown aetiologies (and if conventional gastroenterological therapies have been ineffective) should be examined by echocardiography, and Heyde’s syndrome should be ruled out. Attention should be given to the two main symptoms of Heyde’s syndrome during its diagnosis: severe AS and refractory GIB. The possibility of this disease should not simply be ruled out on the sole basis of the absence of evidence of angiodysplasia or AVWS, but other disorders leading to GIB should also be excluded during diagnostic testing. Local enterectomy could be a bridging therapy for aortic valve replacement in severe hemorrhagic patients. TAVI may be beneficial for moderate to high surgical-risk patients of Heyde’s syndrome regardless of the potential risk of GIB.

## Electronic supplementary material

Below is the link to the electronic supplementary material.

Additional file 1: Summary of case reports of Heyde’s syndrome

## Data Availability

The datasets of the current study are available from the corresponding author upon reasonable request.

## Abbreviations

AS: Aortic valve stenosis
GIB: Gastrointestinal bleeding
HMWM-vWFs: High-molecular-weight multimers of vWFs
AVWS: Acquired von Willebrand syndrome
LVOTO: Left ventricular outflow tract obstruction
TAVI: Transcatheter aortic valve implantation
Hb: Hemoglobin
TTE: Transthoracic echocardiography
HCM: Hypertrophic cardiomyopathy
SAVR: Surgical aortic valve replacement
LVOT: Left ventricular outflow tract
PPIs: Proton pump inhibitors
