# Supplementary material for: Did angiodysplasia associated with heyde’s syndrome disappear spontaneously?: a case report
Source: J Cardiothorac Surg. 2023 Jul 10;18:225. doi: 10.1186/s13019-023-02337-8 (PMC10334555; doi:10.1186/s13019-023-02337-8)
Supplement: Supplementary file 1 — Additional file 1: Summary of case reports of Heyde’s syndrome [file 13019_2023_2337_MOESM1_ESM.doc]

| **Supplementary table 1.** Summary of case reports of Heyde’s syndrome | | | | | | | | | | |
| --- | --- | --- | --- | --- | --- | --- | --- | --- | --- | --- |
| Reference No. | Case No. | Age | Sex | Primary Disease | Therapy | Pretherapy | | | Post-treatment | |
|  |  |  |  |  |  | Bleeding episodes | Angiodysplasia | HMWM-vWFs deficiency | Bleeding episodes | Follow-up time(months ) |
| 1 | 1 | 85 | female | AS | Medications | ＋ | ＋ | ＋ | Death | — |
| 2 | 2 | 83 | female | AS | TAVI | ＋ | ＋ | ＋ | 0 | 48 |
| 3 | 3 | 82 | female | AS | Medications | ＋ | ＋ | 0 | 0 | — |
| 4 | 4 | 72 | female | AS | Endoscopic hemostasis | ＋ | ＋ | — | 0 | 12 |
| 5 | 5 | 61 | male | AS | TAVI | ＋ | 0 | ＋ | 0 | 36 |
| 6 | 6 | 79 | male | AS | TAVR | ＋ | 0 | — | 0 | — |
| 7 | 7 | 42 | male | AS | Medications | ＋ | ＋ | ＋ | 0 | 12 |
| 8 | 8 | 64 | male | AS | SAVR | ＋ | ＋ | — | 0 | — |
| 9 | 9 | 85 | male | AS | TAVI | ＋ | ＋ | 0 | 0 | 6 |
| 10 | 10 | 56 | female | AS | TAVR | ＋ | 0 | ＋ | ＋ | 6 |
| 11 | 11 | 77 | female | AS | SAVR | ＋ | ＋ | ＋ | 0 | 1 |
| 12 | 12 | 87 | male | AS | SAVR | ＋ | ＋ | ＋ | ＋ | 20 |
| 13 | 13 | 81 | male | AS | TAVI | ＋ | ＋ | — | 0 | 6 |
| 14 | 14 | 67 | female | AS | SAVR | ＋ | ＋ | ＋ | 0 | 6 |
| 15 | 15 | 82 | male | AS | TAVI | ＋ | ＋ | ＋ | 0 | 12 |
| 16 | 16 | 67 | female | HOCM | ASA | ＋ | 0 | — | 0 | 12 |
| 17 | 17 | 70 | male | AS | SAVR | ＋ | ＋ | — | 0 | 2 |
| 18 | 18 | 67 | male | AS | SAVR | ＋ | ＋ | — | 0 | 6 |
| 19 | 19 | 71 | male | AS | SAVR | ＋ | ＋ | — | 0 | — |
| 20 | 20 | 62 | female | AS | SAVR | ＋ | ＋ | — | 0 | — |
| 21 | 21 | 75 | female | AS | TAVI | ＋ | ＋ | — | 0 | 6 |
| 22 | 22 | 76 | male | AS | Medications | ＋ | ＋ | — | Death | — |
| 23 | 23 | 64 | female | AS | SAVR | ＋ | ＋ | — | 0 | — |
| 24 | 24 | 78 | female | AS | SAVR | ＋ | ＋ | ＋ | 0 | 0.25 |
| **continued** | | | | | | | | | | |
| 25 | 25 | 51 | female | AS | Medications | ＋ | 0 | — | Death | — |
| 26 | 26 | 64 | male | AS | Segmental enterectomy | ＋ | ＋ | ＋ | 0 | 12 |
| 27 | 27 | 77 | male | AS | SAVR | ＋ | ＋ | — | ＋ | 3 |
| 28 | 28 | 57 | male | HOCM | ASA | ＋ | 0 | — | ＋ | 2 |
| 29 | 29 | 64 | female | HOCM | Endoscopic hemostasis | ＋ | ＋ | — | 0 | 12 |
| 30 | 30 | 84 | female | AS | TAVI | ＋ | ＋ | ＋ | 0 | — |
| 31 | 31 | 50 | male | AI | SAVR | ＋ | ＋ | ＋ | 0 | 1 |
| 32 | 32 | 80 | female | AS | TAVI | ＋ | ＋ | — | 0 | 3 |
| 33 | 33 | 70 | male | AS | SAVR | ＋ | ＋ | ＋ | Death | — |
| 34 | 34 | 73 | female | AS | TAVI | ＋ | 0 | ＋ | 0 | 3 |
| 35 | 35 | 56 | female | AS | SAVR | ＋ | ＋ | — | 0 | — |
| 36 | 36 | 63 | female | AS | SAVR | ＋ | 0 | — | 0 | — |
| 37 | 37 | 84 | female | AS | TAVI | ＋ | ＋ | — | 0 | 4 |
| 38 | 38 | 76 | female | AS | TAVI | ＋ | 0 | ＋ | 0 | — |
| 39 | 39 | 82 | male | AS | TAVI | ＋ | ＋ | ＋ | 0 | 12 |
| 40 | 40 | 61 | male | AS | — | ＋ | ＋ | — | — | — |
| 41 | 41 | 70 | female | HOCM | Medications | ＋ | ＋ | ＋ | 0 | 1 |
| 42 | 42 | 80 | female | AS | SAVR | ＋ | ＋ | — | 0 | 6 |
| 43 | 43 | 46 | female | AS | SAVR | ＋ | ＋ | — | 0 | 12 |
| 44 | 44 | 68 | male | AS | SAVR | ＋ | ＋ | 0 | 0 | 1 |
| 45 | 45 | 72 | male | AS | Embolization of mesenteric artery | ＋ | ＋ | — | 0 | 48 |
| 46 | 46 | 77 | male | AS | TAVI | ＋ | ＋ | ＋ | 0 | 10 |
| 47 | 47 | 76 | female | AS | SAVR | ＋ | — | — | 0 | 4 |
| 48 | 48 | 75 | female | AS | — | ＋ | ＋ | — | — | — |
| 49 | 49 | 76 | female | AS | SAVR | ＋ | ＋ | — | 0 | 42 |
| 50 | 50 | 93 | female | AS | Medications | ＋ | 0 | — | Death | — |
| **continued** | | | | | | | | | | |
| 51 | 51 | 77 | female | AS | SAVR | ＋ | 0 | — | 0 | — |
| 52 | 52 | 79 | male | AS | SAVR | ＋ | ＋ | — | 0 | 108 |
| 53 | 53 | 66 | male | AS | Refusing treaments | ＋ | ＋ | — | — | — |
| 54 | 54 | 89 | female | AS | Refusing treaments | ＋ | ＋ | 0 | — | — |
| 55 | 55 | 79 | male | AS | SAVR | ＋ | ＋ | ＋ | 0 | 3 |
| 56 | 56 | 69 | male | AS | SAVR | ＋ | ＋ | — | 0 | — |
| 57 | 57 | 82 | female | AS | SAVR | ＋ | ＋ | ＋ | 0 | 2 |
| 58 | 58 | 82 | female | AS | Medications | ＋ | ＋ | ＋ | 0 | 6 |
| 59 | 59 | 74 | female | AS | SAVR | ＋ | ＋ | 0 | 0 | 7 |
| 60 | 60 | 68 | female | AS | Endoscopic hemostasis | ＋ | ＋ | — | 0 | — |
| 61 | 61 | 90 | female | AS | Endoscopic hemostasis | ＋ | ＋ | — | 0 | — |
| 62 | 62 | 71 | male | AS | Refusing treaments | ＋ | ＋ | — | — | — |
| 63 | 63 | 85 | male | AS | Refusing treaments | ＋ | 0 | ＋ | — | — |
|  | 64 | 83 | female | AS | Refusing treaments | ＋ | 0 | ＋ | — | — |
| 64 | 65 | 58 | male | AS | Medications | ＋ | ＋ | — | 0 | 25 |
| 65 | 66 | 71 | female | AS | SAVR | ＋ | 0 | 0 | 0 | 6 |
|  | 67 | 83 | male | AS | Medications | ＋ | ＋ | — | — | — |
| 66 | 68 | 70 | male | AS | SAVR | ＋ | ＋ | — | ＋ | 10 |
| 67 | 69 | 67 | female | HOCM | Segmental enterectomy | ＋ | ＋ | — | 0 | — |
|  | 70 | 78 | female | HOCM | Medications | ＋ | ＋ | — | — | — |
| 68 | 71 | 83 | female | AS | Medications | ＋ | ＋ | — | 0 | — |
| 69 | 72 | 68 | female | AS | Endoscopic hemostasis | ＋ | ＋ | ＋ | 0 | — |
| 70 | 73 | 73 | male | AS | Endoscopic hemostasis | ＋ | ＋ | 0 | 0 | 9 |
|  | 74 | 57 | male | AS | Medications | ＋ | ＋ | 0 | Death | — |
| 71 | 75 | 65 | female | AS | SAVR | ＋ | ＋ | ＋ | 0 | 120 |
|  | 76 | 70 | female | AS | SAVR | ＋ | ＋ | ＋ | 0 | 120 |
| 72 | 77 | 61 | male | AS | SAVR | ＋ | ＋ | — | 0 | — |
| **Continued** | | | | | | | | | | |
| 73 | 78 | 77 | male | AS | SAVR | ＋ | 0 | ＋ | 0 | 20 |
| 74 | 79 | 86 | female | AS | TAVI | ＋ | 0 | — | 0 | 3 |
| 75 | 80 | 89 | female | AS | TAVI | ＋ | ＋ | — | 0 | 6 |
| 76 | 81 | 83 | female | AS | TAVI | ＋ | ＋ | — | 0 | — |
| 77 | 82 | 68 | male | AS | SAVR | ＋ | ＋ | 0 | 0 | 9 |
| 78 | 83 | 75 | male | AS | SAVR | ＋ | ＋ | — | 0 | — |
| SAVR: Surgical Aortic Valve Replacement; TAVI: Transcatheter Aortic Valve Implantation; AS: Aortic Valve Stenosis; AI: Aortic valve Insufficiency; HOCM: Hypertrophic Obstructive Cardiomyopathy; ASA: Alcohol Septal Ablation;(+)：yes;(0):no;(—):not available  **Reference:**  [1]SINHA S, CASTRO D, SHAKIL S. Medical Management of Heyde Syndrome[J]. Cureus， 2021，13(1):e12551.  [2]TSUCHIYA S, MATSUMOTO Y, DOMAN T, et al. Disappearance of Angiodysplasia Following Transcatheter Aortic Valve Implantation in a Patient with Heyde's Syndrome: A Case Report and Review of the Literature[J]. J Atheroscler Thromb， 2020，27(3):271-7.  [3]THAKKER RA, SUTHAR KH, KLINE K, et al. Aortic Stenosis Complicated by Gastrointestinal Arteriovenous Malformations: It is not Always Heyde Syndrome[J]. Cureus， 2020，12(6):e8876.  [4]NOOR A, ABADCO D. Heyde Syndrome Complicated by a Dieulafoy Lesion[J]. The Ochsner journal， 2020，20(3):326-30.  [5]CHUKWUDUM CA, VERA S, SHARMA M, et al. Heyde Syndrome: A Case Report and Literature Review[J]. Cureus， 2020，12(4):e7896.  [6]AHMED T, HAQUE R. Heyde's Syndrome Complicating Management in a Patient With High Bleeding and Thrombotic Risks[J]. Cureus， 2020，12(5):e8280.  [7]SCHWAIGER JP, LUDWICZEK O, GRAZIADEI I, et al. A Vicious Circle: Heyde Syndrome in Mild Aortic Stenosis[J]. CASE (Philadelphia, Pa)， 2019，3(4):171-6.  [8]GARCIA LR, GARZESI AM, TRIPOLI G, et al. Heyde Syndrome Treated by Conventional Aortic Valve Replacement[J]. Braz J Cardiovasc Surg， 2019，34(5):630-2.  [9]RAMACHANDRAN R, UQDAH H, JANI N. A case of recurrent obscure gastrointestinal bleeding: Heyde's syndrome - case report and review[J]. J Community Hosp Intern Med Perspect， 2018，8(3):127-9.  [10]ALSHUWAYKH O, KRIER MJ. A Case of Heyde Syndrome with Resolution of Gastrointestinal Bleeding Two Weeks After Aortic Valve Replacement[J]. The American journal of case reports， 2018，19:924-6.  [11]UCHIDA T, HAMASAKI A, OHBA E, et al. Life-threatening subdural hematoma after aortic valve replacement in a patient with Heyde syndrome: a case report[J]. J Cardiothorac Surg， 2017，12(1):65.  [12]SHIBAMOTO A, KAWARATANI H, KUBO T, et al. Aortic Valve Replacement for the Management of Heyde Syndrome: A Case Report[J]. J Nippon Med Sch， 2017，84(4):193-7.  [13]BALBO CP, SEABRA LP, GALORO VG, et al. Heyde's Syndrome and Transcatheter Aortic Valve Implantation[J]. Arq Bras Cardiol， 2017，108(4):378-80.  [14]AKUTAGAWA T, SHINDO T, YAMANOUCHI K, et al. Persistent Gastrointestinal Angiodysplasia in Heyde's Syndrome after Aortic Valve Replacement[J]. Intern Med， 2017，56(18):2431-3.  [15]HUDZIK B, WILCZEK K, GASIOR M. Heyde syndrome: gastrointestinal bleeding and aortic stenosis[J]. Cmaj， 2016，188(2):135-8.  [16]HVID-JENSEN HS, POULSEN SH, AGNHOLT JS. Severe Gastrointestinal Bleeding in a Patient With Subvalvular Aortic Stenosis Treated With Thalidomide and Octreotide: Bridging to Transcoronary Ablation of Septal Hypertrophy[J]. J Clin Med Res， 2015，7(11):907-10.  [17]KAPILA A, CHHABRA L, KHANNA A. Valvular aortic stenosis causing angiodysplasia and acquired von Willebrand's disease: Heyde's syndrome[J]. BMJ case reports， 2014，2014  [18]CAPUANO F, ANGELONI E, ROSCITANO A, et al. Blackish Pigmentation of the Aorta in Patient with Alkaptonuria and Heyde's Syndrome[J]. Aorta (Stamford, Conn)， 2014，2(2):74-6.  [19]DOS SANTOS VM, DOS SANTOS LA, MODESTO AA, et al. Heyde syndrome in a 71-year-old man who underwent chest radiotherapy at young age[J]. Anales del sistema sanitario de Navarra， 2013，36(2):339-45.  [20]MARTíNEZ-CASELLES A, MARTíNEZ-PASCUAL C, SáNCHEZ-TORRES A, et al. Beyond Heyde's syndrome[J]. Revista espanola de enfermedades digestivas : organo oficial de la Sociedad Espanola de Patologia Digestiva， 2012，104(11):615-6.  [21]GüL M, SüRGIT Ö, ÖZAL E, et al. Treatment of aortic valve stenosis and gastrointestinal bleeding by transcatheter aortic valve implantation in Heyde syndrome[J]. Anadolu Kardiyol Derg， 2012，12(8):691-3.  [22]FIGUINHA FC, SPINA GS, TARASOUTCHI F. Heyde's syndrome: case report and literature review[J]. Arq Bras Cardiol, 2011,96(3):e42-5.  [23]RAHHAL F, CHAMBERLAIN S. Education and Imaging. Gastrointestinal: Heyde's syndrome[J]. Journal of gastroenterology and hepatology， 2009，24(6):1150.  [24]MORISHIMA A, MARUI A, SHIMAMOTO T, et al. Successful aortic valve replacement for Heyde syndrome with confirmed hematologic recovery[J]. Ann Thorac Surg， 2007，83(1):287-8.  [25]GARCíA FANJUL RM, ANTUñA BRAñA MT, LACORT FERNáNDEZ M. [Commentary on a case of Heyde's syndrome][J]. Anales de medicina interna (Madrid, Spain : 1984)， 2007，24(12):612-3.  [26]OGANO M, IWASAKI YK, TAKANO H, et al. Successful colectomy for the treatment of repetitive bleeding from colonic angiodysplasia in a patient with Heyde syndrome[J]. Intern Med， 2006，45(6):355-8.  [27]GIUSTI DE MARLE M, SGRECCIA A, CARMENINI E, et al. Infective endocarditis from Enterococcus faecalis complicating colonoscopy in Heyde's syndrome[J]. Postgraduate medical journal， 2004，80(948):619-20.  [28]RIIS HANSEN P, HASSAGER C. Septal alcohol ablation and Heyde's syndrome revisited[J]. Journal of internal medicine， 2003，253(4):490-1.  [29]FUJITA H, TOMIYAMA J, CHUGANJI Y, et al. Diffuse angiodysplasia of the upper gastrointestinal tract in a patient with hypertrophic obstructive cardiomyopathy[J]. Intern Med， 2000，39(5):385-8.  [30]ABE D, ASAOKA D, IKEDA A, et al. [Heyde syndrome successfully treated with transcatheter aortic valve implantation to prevent recurrence of gastrointestinal bleeding from angiodysplasia][J]. Nihon Shokakibyo Gakkai zasshi = The Japanese journal of gastro-enterology， 2020，117(9):802-10.  [31]KASAI M, OSAKO M, INABA Y, et al. Acquired von Willebrand syndrome secondary to mitral and aortic regurgitation[J]. J Card Surg， 2020，35(9):2396-8.  [32]FAMULARO G, MARROLLO M. Of aortic valve and bleeding: Heyde's syndrome[J]. The American journal of emergency medicine， 2020，38(11):2493.e1-.e2.  [33]OTANI T, HATAKEYAMA K, YAMASHITA K, et al. A histological description of intestinal 'angiodysplasia' in an autopsy case of Heyde's syndrome[J]. Pathol Int， 2019，69(12):727-9.  [34]MIRNA M, LICHTENAUER M, THEURL T, et al. Transcatheter aortic valve implantation in a patient with suspected hereditary von Willebrand disease and severe gastrointestinal bleeding - a case report[J]. Scott Med J， 2019，64(4):142-7.  [35]MAHBOOBI SK. Heyde's syndrome and postoperative bleeding after aortic valve replacement - Is there a role for prophylactic desmopressin?[J]. J Clin Anesth， 2019，56:142.  [36]MILLA M, HERNáNDEZ E, MéRIDA E, et al. Heyde syndrome: Correction of anemia after aortic valve replacement in a hemodialysis patient[J]. Nefrologia (Engl Ed)， 2018，38(3):327-9.  [37] Wang Bin, Wang Yan, Su Maolong. Case report of a patient with Heyde syndrome treated by transcatheter aortic valve implantation. Chin J Cardiol 2016, 44(3): 269-270.  [38] Huang Dajun, Kong Lingqiu, Wu Zhou. A case of Heyde syndrome: resolution following transcatheter aortic valve implantation. Chin J Intern Med 2016, 55(9): 721-722.  [39] Yang Yujing, Xu Chengyi. Hyde syndrome treated by transcatheter aortic valve replacement：a case report. Chin J Cardiovasc Med 2021;26:183-185.  [40]ALSIDAWI S, COUTO M, LóPEZ-CANDALES A. Acquired Von Willebrand Syndrome In Aortic Stenosis: Case Report And Review[J]. Boletin de la Asociacion Medica de Puerto Rico， 2015，107(2):86-8.  [41]WAKE M, TAKAHASHI N, YOSHITOMI H, et al. A case of hypertrophic obstructive cardiomyopathy and acquired von Willebrand syndrome: response to medical therapy[J]. Journal of echocardiography， 2014，12(3):112-4.  [42]TAGUCHI T, WATANABE M, WATADANI K, et al. A case of Heyde syndrome: resolution following aortic valve replacement[J]. The heart surgery forum， 2014，17(5):E258-60.  [43]İLKELI E, ALBEYOĞLU Ş, ÇILOĞLU U, et al. Heyde's syndrome[J]. Asian cardiovascular & thoracic annals， 2014，22(5):592-4.  [44]GODINHO AR, AMORIM S, CAMPELO M, et al. Severe aortic stenosis: forgotten associations[J]. Revista portuguesa de cardiologia : orgao oficial da Sociedade Portuguesa de Cardiologia = Portuguese journal of cardiology : an official journal of the Portuguese Society of Cardiology， 2014，33(9):563.e1-4.  [45]FAMULARO G, GASBARRONE L, MINISOLA G. Octreotide for Heyde's syndrome: a case report[J]. Presse Med， 2014，43(11):1281-3.  [46]BENTON SM, JR., KUMAR A, CRENSHAW M, et al. Effect of transcutaneous aortic valve implantation on the Heyde's syndrome[J]. Am J Cardiol， 2014，114(6):953-4.  [47]SAAD RA, LWALEED BA, KAZMI RS. Gastrointestinal bleeding and aortic stenosis (Heyde syndrome): the role of aortic valve replacement[J]. J Card Surg， 2013，28(4):414-6.  [48]NEGI SI, XU R, ANAND A. Medical image. A murmur and a bleed: the Heyde syndrome[J]. The New Zealand medical journal， 2013，126(1371):95-7.  [49]MAOR NR. Heyde syndrome: resolution of anemia after aortic valve surgery[J]. The Israel Medical Association journal : IMAJ， 2013，15(7):387-9.  [50]LEDINGHAM D. Heyde's syndrome: exploring the link between aortic stenosis and an acquired bleeding disorder[J]. BMJ case reports， 2013，2013  [51]MORAN GW, BRIND A. Anaemia in a cardiopath[J]. Gut， 2012，61(4):513, 81.  [52]MICHOT JM, TRETON X, BRINK C, et al. Severe gastro-intestinal angiodysplasia in context of Heyde's syndrome durably cured after aortic valve replacement[J]. Presse Med， 2012，41(7-8):763-6.  [53]ISLAM S, ISLAM E, CEVIK C, et al. Aortic stenosis and angiodysplastic gastrointestinal bleeding: Heyde's disease[J]. Heart & lung : the journal of critical care， 2012，41(1):90-4.  [54]GARCíA-MARTíN A, MORENO A, MORO C. Heyde's Syndrome[J]. Revista espanola de cardiologia, 2011，64(1):75-7.  [55]D'SOUZA PM, BLOSTEIN MD. Diagnosis of Heyde's syndrome by abnormal closure times despite normal von Willebrand's activity[J]. Blood coagulation & fibrinolysis : an international journal in haemostasis and thrombosis， 2011，22(7):622-3.  [56]VAZ A, CORREIA A, MARTINS B, et al. Heyde syndrome--the link between aortic stenosis and gastrointestinal bleeding[J]. Revista portuguesa de cardiologia : orgao oficial da Sociedade Portuguesa de Cardiologia = Portuguese journal of cardiology : an official journal of the Portuguese Society of Cardiology， 2010，29(2):309-14.  [57]TAKAHASHI N, TANABE K, YOSHITOMI H, et al. Successful endoscopic clipping for bleeding from colonic angiodysplasia in a case of Heyde syndrome[J]. Medical science monitor : international medical journal of experimental and clinical research， 2010，16(9):Cs107-9.  [58]GANDHI V, PHILIP S, NAGRAL S. Heyde syndrome[J]. Tropical gastroenterology : official journal of the Digestive Diseases Foundation， 2010，31(2):120-1.  [59]UNDAS A, WINDYGA J, BYKOWSKA K, et al. Heyde's syndrome without a decrease in large von Willebrand factor multimers: a case of intestinal bleedings reversed by valve replacement in a patient with aortic stenosis[J]. Thromb Haemost， 2009，101(4):773-4.  [60]HUI YT, LAM WM, FONG NM, et al. Heyde's syndrome: diagnosis and management by the novel single-balloon enteroscopy[J]. Hong Kong medical journal = Xianggang yi xue za zhi， 2009，15(4):301-3.  [61]HOKAMA A, KISHIMOTO K, HIGASHIARAKAWA M, et al. Heyde syndrome: a common but less recognized complex of aortic stenosis and bleeding intestinal angiodysplasia[J]. Southern medical journal， 2009，102(12):1279.  [62]FLOUDAS CS, MOYSSAKIS I, PAPPAS P, et al. Obscure gastrointestinal bleeding and calcific aortic stenosis (Heyde's syndrome)[J]. Int J Cardiol， 2008，127(2):292-4.  [63]SCHöDEL J, OBERGFELL A, MAASS AH. Severe aortic valve stenosis and nosebleed[J]. Int J Cardiol， 2007，120(2):286-7.  [64]DE PALMA GD, SALVATORI F, MASONE S, et al. Acute gastrointestinal bleeding following aortic valve replacement in a patient with Heyde's sindrome. Case report[J]. Minerva gastroenterologica e dietologica， 2007，53(3):291-3.  [65]PERISIĆ N, DODER R, ILIĆ R, et al. [Heyde's syndrome][J]. Vojnosanitetski pregled， 2006，63(7):673-6.  [66]GIOVANNINI I, CHIARLA C, MURAZIO M, et al. An extreme case of Heyde syndrome[J]. Digestive surgery， 2006，23(5-6):387-8.  [67]MARTí L, ANTóN R, ALMELA P, et al. [Intestinal angiodysplasia and hypertrophic subaortic stenosis: is it a Heyde's syndrome variant?][J]. Medicina clinica， 2005，125(16):635-6.  [68]CORRêA PL, FELIX RC, AZEVEDO JC, et al. Gastrointestinal bleeding diagnosed by red blood cell scintigraphy in a patient with aortic stenosis: a case of Heyde syndrome[J]. Clinical nuclear medicine， 2005，30(4):231-5.  [69]LEE TY, HAN SY, MOON SH, et al. [A case of Heyde's syndrome with abnormal von Willebrand factor][J]. The Korean journal of gastroenterology = Taehan Sohwagi Hakhoe chi， 2004，43(2):133-6.  [70]KARAJEH MA, MCALINDON ME, MAKRIS M, et al. Bleeding small bowel angiodysplasia in association with aortic stenosis: a role for combined wireless capsule endoscopy and push enteroscopy?[J]. J Clin Gastroenterol， 2004，38(9):836-7.  [71]WARKENTIN TE, MOORE JC, MORGAN DG. Gastrointestinal angiodysplasia and aortic stenosis[J]. N Engl J Med， 2002，347(11):858-9.  [72]GRANEL B, SERRATRICE J, BERNIT E, et al. [Heyde syndrome][J]. Presse Med， 2002，31(31):1451-3.  [73]IIJIMA M, ITOH N, MURASE R, et al. A surgical case of aortic stenosis with recurrent gastrointestinal bleeding: Heyde syndrome[J]. Int J Surg Case Rep， 2018，53:281-4.  [74]RASHID S, MALKIN C, SCHLOSSHAN D, et al. Severe aortic stenosis, critical coronary artery disease, and transfusion-dependent angiodysplasia - A management conundrum[J]. J Cardiol Cases， 2016，13(3):87-9.  [75]PYXARAS SA, SANTANGELO S, PERKAN A, et al. Reversal of angiodysplasia-derived anemia after transcatheter aortic valve implantation[J]. J Cardiol Cases， 2012，5(2):e128-e31.  [76]GODINO C, PAVON AG, MANGIERI A, et al. Aortic Valvuloplasty as Bridging for TAVI in High-Risk Patients with Heyde's Syndrome: A Case Report[J]. Case Rep Med， 2012，2012:946764.  [77]ABI-AKAR R, EL-RASSI I, KARAM N, et al. Treatment of Heyde's Syndrome by Aortic Valve Replacement[J]. Current cardiology reviews， 2011，7(1):47-9.  [78]OHTA S, WATANABE T, MORITA S, et al. Massive jejunal bleeding due to Heyde syndrome successfully treated with double balloon endoscopy[J]. Clinical journal of gastroenterology， 2009，2(3):187-9. | | | | | | | | | | |
